# Challenges and Opportunities in Big Data Science to Address Health Inequities and Focus the HIV Response

**DOI:** 10.1007/s11904-024-00702-3

**Published:** 2024-06-25

**Authors:** Katherine Rucinski, Jesse Knight, Kalai Willis, Linwei Wang, Amrita Rao, Mary Anne Roach, Refilwe Phaswana-Mafuya, Le Bao, Safiatou Thiam, Peter Arimi, Sharmistha Mishra, Stefan Baral

**Affiliations:** 1 Department of International Health, Johns Hopkins School of Public Health, Baltimore, MD USA; 2 MAP Centre for Urban Health Solutions, Unity Health Toronto, Toronto, ON Canada; 3 Institute of Medical Science, University of Toronto, Toronto, ON Canada; 4 Department of Epidemiology, Johns Hopkins School of Public Health, Baltimore, MD USA; 5 South African Medical Research Council/University of Johannesburg Pan African Centre for Epidemics Research (PACER) Extramural Unit, Johannesburg, South Africa; 6 Department of Environmental Health, Faculty of Health Sciences, University of Johannesburg, Johannesburg, South Africa; 7 Department of Statistics, Pennsylvania State University, University Park, PA USA; 8 Conseil National de Lutte Contre Le Sida, Dakar, Senegal; 9 Partners for Health and Development in Africa, Nairobi, Kenya; 10 Division of Infectious Diseases, Department of Medicine, University of Toronto, Toronto, ON Canada; 11 Institute of Health Policy, Management and Evaluation & Division of Epidemiology, Dalla Lana School of Public Health, University of Toronto, Toronto, ON Canada; 12 ICES, Toronto, ON Canada

**Keywords:** Big Data Science, HIV transmission dynamics, Health equity, Community HIV response, Key populations, Predictive modeling, Explanatory modeling

## Abstract

**Purpose of Review:**

Big Data Science can be used to pragmatically guide the allocation of resources within the context of national HIV programs and inform priorities for intervention. In this review, we discuss the importance of grounding Big Data Science in the principles of equity and social justice to optimize the efficiency and effectiveness of the global HIV response.

**Recent Findings:**

Social, ethical, and legal considerations of Big Data Science have been identified in the context of HIV research. However, efforts to mitigate these challenges have been limited. Consequences include disciplinary silos within the field of HIV, a lack of meaningful engagement and ownership with and by communities, and potential misinterpretation or misappropriation of analyses that could further exacerbate health inequities.

**Summary:**

Big Data Science can support the HIV response by helping to identify gaps in previously undiscovered or understudied pathways to HIV acquisition and onward transmission, including the consequences for health outcomes and associated comorbidities. However, in the absence of a guiding framework for equity, alongside meaningful collaboration with communities through balanced partnerships, a reliance on big data could continue to reinforce inequities within and across marginalized populations.

## Introduction

### Increasing the Specificity of the HIV Response Means Explicitly Identifying and Addressing Health Inequities

We are at a pivotal moment in the global HIV response. Reductions in HIV incidence are at risk of losing momentum given a failure to address systematic gaps in prevention and treatment. HIV incidence is at a thirty-year low, yet 1.5 million people still acquire HIV each year, including about one million in countries across Sub-Saharan Africa [1]. Investments in HIV programming, along with advances in treatment and prevention, including antiretroviral therapy (ART) and pre-exposure prophylaxis (PrEP), have the potential to reduce transmission [2–5], but marked differences in access are limiting global progress and amplifying health inequities [6•]. Widespread ART rollout through national HIV programs has improved the availability of treatment for people living with HIV. However, only 68% of the estimated 38.4 million people living with HIV globally have been able to achieve viral suppression [1]. Further, despite large, multi-sector investments in PrEP scale-up over the last five years [7, 8], uptake and persistence have been lower than expected globally, and especially among individuals at high risk of HIV [9, 10].

Underlying gaps in treatment and prevention is the acknowledgement that HIV risks are not evenly distributed anywhere in the world, with heterogeneity in HIV acquisition and onward transmission well-established through both empirical studies and mathematical models [11•, 12]. This heterogeneity often reflects complex sexual and/or shared injecting networks that intersect with social, political, and economic marginalization [6•, 13•]. While heterogeneity in HIV acquisition has largely been used to guide the population-level interventions and national HIV programs that define the HIV response, heterogeneity in onward transmission has not been as consistently considered. This is particularly true in settings with a high prevalence of HIV, where the epidemic is largely considered “generalized”, such as across countries in eastern and southern Africa [6•, 13•]. When there is also heterogeneity in onward transmission risks, health inequities are amplified if those who are most at risk of onward transmission are also least likely to be reached by programs and services [14–17•]. Understanding the intersections of heterogeneity in risk and heterogeneity in intervention coverage is fundamental to addressing health inequities within epidemics.

The current mismatch between the realities of the HIV pandemic and the existing response could continue to reinforce inequities within and across marginalized communities, ultimately undermining the progress made on the path to ends AIDS. In the context of the HIV epidemic, inequalities refer to heterogeneity in measures of: HIV acquisition, ART coverage, viral suppression, and PrEP uptake, along with other measures of prevention coverage. When these inequalities stem from preventable and modifiable mechanisms, they reflect larger systemic health inequities [18]. One such preventable mechanism relates to intervention access, including barriers within existing health-systems such as stigma and discrimination, institutionalized racism, homophobia, transphobia, ageism, and sexism. Communities most affected by these barriers are those that have been disproportionately impacted by HIV, including the LGBTQ community, sex workers, and people who inject drugs, among others. Thus, an equity-informed strategy for a given intervention, such as HIV treatment to achieve sustained viral suppression, involves addressing access barriers within health-systems and “meeting people where they are” with differentiated service delivery [19, 20].

Health equity is a priority for global public health [18, 21]. There are several frameworks for examining health equity in the context of public health. Chief among them is the health equity framework proposed by Peterson et al. which centers health outcomes at a population-level and across four spheres of influence. These spheres comprise relationships and networks, systems of power that determine access to resources, physiological pathways, and individual factors that shape one’s response to their environment [22•]. The first two spheres, networks and systems of power, are particularly important in the context of infectious diseases. First, preventable mechanisms such as criminalization of sex work and barriers to access, can shape the networks within which transmission occurs. Second, preventable mechanisms can also act through systems of power, including decisions around resource allocation and power imbalances in the production policies that are informed by epidemiologic evidence. In the context of the HIV response, systems of power are particularly salient given four decades of community-engagement alongside community-based and community-led HIV research [23]. In addition to the health equity framework [22•], the modified socio-ecological model of HIV prevention and related multi-dimensional conceptual frameworks have also provided a foundation for examining and addressing heterogeneity in HIV risk [24•, 25]. These latter frameworks are similar to the health equity framework in centering population-level outcomes and networks, but with a greater focus on directed causal pathways that lead to HIV acquisition and to onward transmission. The unifying feature across these established conceptual frameworks is that measures of heterogeneity in onward HIV transmission risks represent downstream consequences of structural determinants manifesting as health inequities.

The application of Big Data Science in the field of HIV has expanded significantly over the last decade alongside machine learning algorithms [26], but often with limited attention to how these approaches may amplify or mitigate existing health inequities if results are used to guide the application of interventions and resources. At its core, Big Data Science comprises high-dimensional data, characterized by the “volume, variety, and velocity” of big data [27], along with the application of advanced statistical techniques and modeling approaches that capitalize on computational capacity for data storage and analytic speed [28]. Several frameworks have been proposed to manage challenges related to the social, ethical, and legal considerations of big data [29–31]. However, efforts to integrate disparate data sources and methodologies that are responsive to HIV epidemic heterogeneities have been less common [32]. The consequences include disciplinary silos within the field of HIV, a lack of meaningful engagement and ownership with and by communities, and potential misinterpretation or misappropriation of analyses that could further exacerbate health inequities.

Advancing the HIV response through Big Data Science therefore means systematically identifying the pathways that lead to health inequalities in HIV acquisition and transmission and aligning interventions to maximize health equity. In this paper, we draw on existing health equity frameworks and a modified socio-ecological model of HIV prevention to outline the potential risks of applying conventional Big Data Science approaches with respect to health equity. We then discuss potential opportunities for equity-informed Big Data Science to work towards a HIV response that is effectively aligned with individual, network, and structural risks that continue to drive HIV acquisition and transmission.

### The Promise of Big Data Science

An effective HIV response requires continual reexamination of our knowledge about the pathways and prevention gaps that shape risks of onward transmission in the short- and long-term. Central to this reexamination are three collaborative principles. First, there is a need to meaningfully ground analyses in the lived experience and expertise of communities, particularly communities who face disproportionate risks of HIV acquisition and onward transmission and sustained barriers to healthcare access. Community leadership has long been enshrined as central to the HIV response, first codified with the Greater Involvement of People Living with HIV published in 1994 [33]. However, as prevention gaps become more and more “concentrated” among the most marginalized communities [34], better integrating the knowledge of these communities into the content and implementation of programs is increasingly central. Second, reexamination requires meaningful engagement with front-line programs and service providers, who are often also on the front-line of data collection. Third, there is a need for collaborations that transcend the disciplinary siloes that have historically guided decisions about funding and resource allocation [35]. This means harnessing the expertise of epidemiologists and social scientists alongside statisticians, computer scientists, and infectious disease modelers, among others.

The promise of Big Data Science in guiding the HIV response lies in its potential to help identify gaps in previously undiscovered or understudied pathways to HIV acquisition and onward transmission, including the consequences for health outcomes and associated comorbidities. Integral to Big Data Science for HIV is the concept that large and diverse data sources [28, 36, 37], can be leveraged to better understand HIV epidemics and the pandemic as a whole. These data sources consist of routinely collected data such as electronic health records and program/clinic registers [38, 39], surveillance data, and auxiliary data sources such as social media and digital data along with traditional research data collected through trials or observational studies. Using a Big Data Science approach, these data sources can support analyses that are critical to the HIV response, including the identification of key groups of individuals and priority geographic areas at high risk of HIV, setting program targets, the evaluations of progress towards goals, and the development of strategies for optimizing the impact of HIV prevention and treatment programs.

### How Conventional Approaches in Big Data Science Could Amplify Health Inequities

There are three primary areas where conventional Big Data Science approaches may potentially undermine health equity including privacy, data biases, and opportunity costs [21].

First, privacy concerns need to be contextualized within legal and policy-environments that criminalize occupations, identities, and dependency including sex work, sexual and gender diverse communities, and people who use drugs [40•]. Privacy concerns also reflect power dynamics wherein data reporting, ownership, and governance moves away from communities and to governments, academics, and international policy-makers and donors. Many emerging data sources, such as routinely collected program data and social media data, are indexed at the individual-level and contain identifiable information. These data can include information about HIV status, as well as stigmatized and/or criminalized behaviors, such as buying and selling sex, same-sex practices, and substance use [41, 42]. Yet, repurposing these data for new analyses, including linkages to other datasets, may not be covered by appropriate oversight, informed consent, or principles of equitable data ownership [43]. Moreover, these data and other data sources not originally collected for the purposes of research may be subject to a lower threshold of regulatory oversight [44]. Thus, efforts to leverage these data may expose already vulnerable individuals to new privacy risks and could thereby erode trust and engagement in care [45]. Ensuring that Big Data Science advances equity and community ownership necessitates careful attention to real and perceived risks of privacy breaches. In the absence of this, Big Data Science could risk perpetuating existing power structures that undermine program effectiveness and potentiates health inequities.

Second, Big Data Science is subject to systematic data biases given its use and integration of existing data that are routinely collected through a range of data platforms. Some data collection is purposeful, with randomized trials or prospective surveys designed to capture detailed data and thereby support specific causal inference or predictive utility. However, most data within Big Data Science comprise observational and repurposed data which may be limited in their design, and which inherently introduce the risk for systemic biases such as selection bias, information bias, and analytic biases such as collider bias among others [46].

For example, household-based surveys may fail to reach key populations due to mobility, precarious housing, or congregate living arrangements such as barracks and brothels [47]. The resulting selection bias from these surveys has been shown to generate downwardly biased estimates of HIV prevalence due to missing data [48]. Similarly, people living with HIV who remain unaware of their HIV status or who have not yet initiated HIV treatment are inherently missing from programmatic records, as are individuals who have engaged in HIV services previously but who have subsequently become “lost to clinic” or “lost to care” [49]. Without tracing studies, confirmatory methods, or other potential adjustment to account for these missing observations, these data may perpetuate more optimistic estimates of ART use and viral suppression [50]. Individuals who are marginalized are also under-represented in social media and social networking data because they are less likely to access mobile phones, the internet and other mobile-based technologies [51, 52]. Even if members of vulnerable populations are reached by surveys, they may not disclose stigmatized and/or criminalized behaviors such as anal sex due to social desirability biases [53–56]. If these selection and reporting biases are ignored, the prevalence of such behaviors from large datasets may be underestimated. Further, when these data sources, with their systematic biases entrenched, comprise a large number of observations that minimize the potential for random error, narrow confidence intervals may artificially reinforce the credibility of these estimates and any resulting inferences [57–60].

Another type of systematic bias can arise in how we use data in algorithms for prediction or analyses for inference. Many big data algorithms, such as random forests, neural networks, and others are designed to identify associations and generate predictions, but are not designed to infer causation [61]. Yet without grounding these predictions in established causal theory, such algorithms are liable to draw erroneous conclusions about drivers of transmission. Specifically, such algorithms may suffer from inappropriate adjustment resulting in collider bias or unmeasured confounding, as well as context-specific limits to generalizability. Together, the downstream effects from these analyses could result in priorities for intervention and/or implementation that are inefficient and misaligned with the needs of marginalized communities [12, 62–65]. Even well-chosen causal inference methods are challenged by common nuances in infectious disease epidemiology, such as spillover effects or “interference”, wherein an individual’s infection status is affected by other individuals’ exposures through network effects [66]. Despite the promise of new machine learning algorithms to generate new insights [67], thorough and rigorous validation procedures are needed to ensure these systems avoid reinforcing pre-existing biases that disproportionately affect marginalized communities [68].

Third, there are potential tradeoffs for the time and resources that must be spent to develop data pipelines, implement new algorithms, and validate emerging data sources. Primary data are often missing among those at the highest risk of HIV acquisition and for transmission in settings with the highest HIV burden [69–71]. This is largely because the same underlying mechanisms (e.g., individual, network, structural) that increase HIV acquisition and transmission risks among marginalized populations challenge the ability to reach them, characterize their HIV burden, and evaluate their unmet HIV prevention and treatment needs [57, 72–75]. Further, while the overall amount of available data is growing in HIV-related research [26], the development of approaches that successfully leverage these data to root out and address health inequities have not kept pace. Simply stated, in its current form, Big Data Science cannot yet overcome missing primary data. And the time and resources spent to explore these algorithms and data gaps may reflect an opportunity cost, when compared with reinvesting in purposeful data collection to fill known data gaps and continued development of more conventional causal inference methodology.

### Using Big Data Science to Focus the HIV Response Requires Collaboration and Engagement with Communities and Programs

In the context of the HIV response, both experimental and observational research data have historically been used to answer specific research questions in controlled settings. These data are less available for key populations and other marginalized communities for whom a reliable sampling frame cannot be constructed [76–78].

Moving forward, supplementing research data with emerging data sources including from programs and community-led monitoring approaches may facilitate the collation of data that is more nuanced and reflective of underlying transmission dynamics. Such sources comprise local data inputs, which can provide critical information regarding real-world uptake and engagement with HIV services and characteristics of ongoing inequities that continue to define the HIV response [11•]. While the systematic collection and collation of surveillance data for key populations have increased over the last decade, the use of routinely collected program data and community-led monitoring has traditionally been less utilized by institutions such as UNAIDS and others tasked with making epidemic projections [20, 79, 80]. For example, program data are routinely collected service-delivery data from implementing partners and community organizations, and may include client files, registries, and reporting indicators to support service management/implementation and evaluate routine program activities. Program data are typically collected and stored individually but reported in aggregate as a metric for appraisal against national service-delivery targets or epidemic transition metrics. Unlike experimental data and observational research studies, program data reflect the realities of day-to-day service delivery, and may often be messy and incomplete [81]. This limitation may inhibit the ability to link programmatic records with unique individuals over time [82, 83]. Moreover, aggregate-level data reported through HIV programs may implicate specific interpretations of HIV metrics [41]. Such challenges may discount these data for researchers and public health practitioners in the absence of local knowledge and contextual information that can inform analytic strategies to mitigate such biases. To mitigate these risks, meaningfully collaboration with communities involved in community-led monitoring provides an opportunity to collect and interpret a range of data sources, ultimately better serving networks of individuals at significant risk of HIV acquisition and transmission.

### How Big Data Science Approaches Can Help Advance Health Equity and Specificity in the HIV Response

**Fig. 1:**
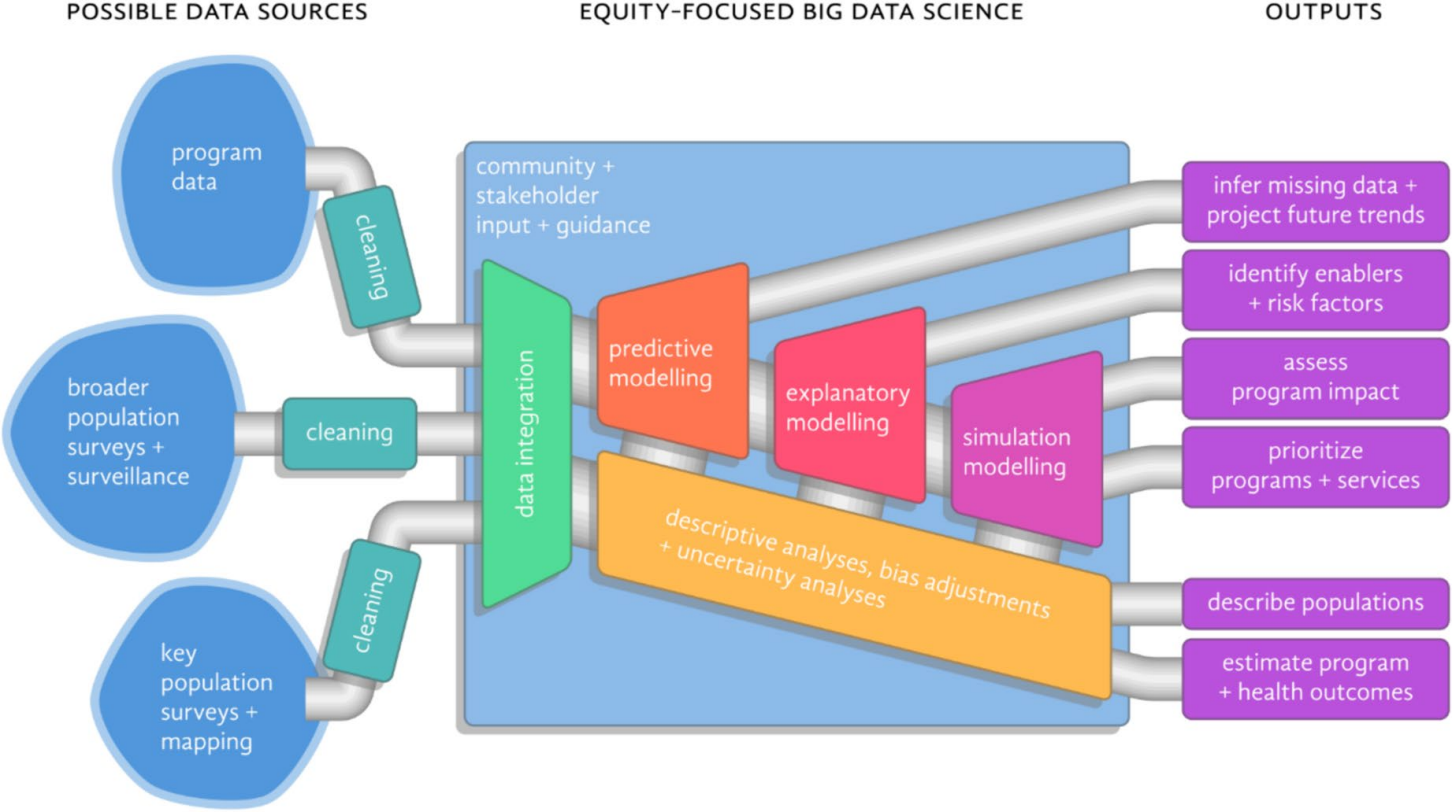
Equity-focused Big Data Science pipeline integrating emerging data sources

Emerging and equity-focused Big Data Science methods have the potential to improve data impact and program efficiency. Such efficiencies are critical for guiding the next phase of the HIV response, particularly given declining available resources from donors and government bodies [84]. These approaches draw on emerging data sources and necessitate investments in data integration, cleaning, and potential bias adjustments to support a range of analytic approaches comprising descriptive, predictive, explanatory, and simulation-based analyses (Fig. 1).

#### Data Integration and Linkage

Optimizing the contributions of emerging data sources in the spirit of informing an equitable HIV response requires attention to both data collation and data integration that will ultimately inform interventions, implementation, and other programmatic decisions. These processes draw on transdisciplinary expertise, whereby researchers, communities, other stakeholders work collaboratively to identify best-practices for combining disparate data sources [85]. Data integration includes three distinct and important elements: data cleaning, linkage, and validation. These integration steps ensure that data are being treated in a systematic and replicable way so that they can be used together and to minimize bias.

HIV-related data, including research data, data from health records, routine program registers, and surveillance systems, are often collected and stored using a wide range of approaches. Data collection may comprise electronic records and paper-based forms. As data are collected there may be real-time updates made to a central data warehouse, or updates to local files requiring manual data entry. Across both approaches, missing data, transcription errors, and incomplete files may result in “noisy” data that necessitate time-consuming cleaning processes [86]. Ultimately, each data file or system will have its own set of rules and challenges, and the process of identifying the rules, addressing the challenges, and harmonizing with an underlying repository is the task of data cleaning.

Once data are cleaned, the next major component to data integration is data linkage. Where unique identifiers are made available, linking records for the same individual over time or across data source types may be feasible [41, 69]. If unique identifiers are imperfectly recorded or unavailable, probabilistic linking algorithms can be utilized to support record linkages. Linking algorithms utilize probabilities of agreement and disagreement between a range of matching variables to link two or more files for the same individual [87]. Compared to a more deterministic approach, probabilistic linking is more forgiving of data entry errors and reporting inconsistencies that may be inherent to non-traditional data sources, particularly routinely-collected program data [88].

As a final step of data integration, data validation involves examining and evaluating the quality of data. The quality of implementation and reporting of research studies, surveillance, and routine program data collection can vary widely, challenging the comparison or integration of these disparate data sources. There are numerous tools and frameworks available to help assess the quality of existing evidence, including the evaluation of randomized controlled trials and cohort studies, measures of disease occurrence, and assessments of internal and external validity. More recent tools have been designed to specifically evaluate the quality of HIV epidemiologic evidence for populations in the absence of a reliable sampling frame, as is often the case for data collected with key populations and other marginalized communities [76]. However, these tools have not been widely implemented in the context of Big Data Science [89]. Data quality should also be assessed with input from front-line data collection teams and community members with lived experience, who can help identify and adjust for context-specific sources of bias and uncertainty.

Overall, integrating data sources allows an assessment of the differential outcomes among people living with HIV who are from historically marginalized communities, as well as provide insights into mitigating these risks. In Malawi, efforts to optimize routine data collected across community-led HIV programs have demonstrated the potential for using real-world data to identify strategies that promote linkage to care and ART uptake for key populations [20, 90]. Importantly, while much of the outreach for marginalized communities is led by community-based organizations, individuals living with HIV are usually referred to government-led treatment clinics upon diagnosis. Supporting effective data integration via intervention strategies that encompass these government-owned data sources would facilitate an understanding of differential outcomes in these clinics and determinants of those who the programs are failing to inform.

#### Description

Gaps in data for marginalized communities can be addressed across a continuum of analytic approaches, with descriptive epidemiology playing a critical role in defining characteristics of a target population across person, places, and time [91]. While causal inference methods draw from a potential outcomes framework to estimate the effect of a proposed intervention, descriptive epidemiology can be used to inform analytic priorities as well as targets for intervention [92]. In the context of Big Data Science, descriptive epidemiology can be useful for working with data sources to understand key elements of a given population, or to document observed or “factual” patterns of disease over time [93]. For example, describing the overall distribution of annualized HIV infections among and across differing identifies or occupations. Importantly, descriptive analyses are still subject to the myriad information and selection biases that plague causal contrasts. However, the manner in which these biases are explored or addressed in the context of Big Data Science has the potential to reinforce inequities in the absence of guidance from community members with context-specific knowledge. For example, overadjustment of key contextual variables such as geography or race can obfuscate important heterogeneity in disease patterns, thus undermining programmatic decisions and priorities for intervention.

#### Prediction

Prediction to inform policy and funding decisions is a central goal of Big Data Science. However, missing data on the most proximal determinants of HIV challenges the predictive accuracy and validity, and thus utility of these approaches. And ignoring proximal determinants in programs can undermine the potential impact of all other efforts at local epidemic control [94]. Thus, while predictive models have long been used to characterize HIV epidemics, including through the use of big data analytics, the underlying disparities in data availability may mean downstream model outputs further perpetuate inequities with consequences especially for marginalized communities [95].

Small area estimation approaches represent a tool in predictive modeling to advance equity in the HIV response. These approaches are less focused on the identification of counterfactuals, and instead are used to characterize epidemiologic patterns in a given place or time. Broadly, small area estimation is a set of statistical techniques used to estimate parameters for “small areas”. These techniques are generally applied when the “small area” of interest is part of a larger survey when empiric estimates may have unacceptably large standard errors or where there are no empiric data. This may be particularly valuable in the case of estimating population size estimates, or other HIV-related outcomes for key populations used to inform program planning and resource allocation for HIV programs. Here, a model-based approach uses a statistical model that “borrows strength” from other small areas or years in the sample survey or from auxiliary data at the small area level. For example, traditional extrapolation approaches to produce estimates where there are no empiric data for key populations relied on crude regression methods that assume homogeneity of all urban centers or rural areas—assumptions that are unlikely to hold true near mining industries, areas with high immigration, settlements along busy roadways, and fishing villages [96, 97]. Moreover, where data do exist, there is limited utilization to inform mathematical modeling and local programmatic or policy response [62, 72, 98, 99]. Efforts to mitigate epidemiologic gaps for marginalized communities have been successful in leveraging social media data to help tackle complex questions around population size in highly stigmatized settings, including as a way of informing direct programs for gay, bisexual, and other cisgender men who have sex with men [100]. Recent research in Namibia has also reinforced the programmatic importance of integrating consensus-building into size estimation methodologies [101], reflecting a larger trend of ensuring community are directly involved in the generation and application of size estimates to maximize relevance for policies and programs [102].

#### Explanatory Modeling

Data may be used to answer explanatory epidemiologic questions, including both descriptive and causal analyses. Whereas descriptive questions aim to characterize features of the target population, causal questions aim to isolate effects of key exposures and/or interventions on specified outcomes [91]. Answering explanatory epidemiologic questions has the potential to help us understand current and relevant implementation challenges, optimize interventions, and improve service delivery. The distinct advantage of using emerging data sources, including routinely collected data, rather than exclusively research data to answer explanatory questions is that even well-designed, community-based studies often do not adequately capture real-world conditions, including available resources, competing priorities of those implementing services or an intervention, and local context [103]. In South Africa, routinely collected data from a community-led HIV program has been successfully used to identify implementation strategies associated with PrEP uptake and persistence within a large cohort of women at high risk of HIV in South Africa [104, 105]. This ability to capture real-world conditions also lends itself to being able to answer additional implementation-related questions. These include assessing the effectiveness of distinct intervention components, fidelity assessments as a mechanism or mediatory of intervention success, as well as factors associated with intervention implementation. Routinely collected data from local program partners harnessed in conjunction with other related data can be used to conduct rigorous epidemiologic and implementation-related analyses to identify impactful interventions across programs for key populations.

#### Simulation Modeling

Compartmental models and agent-based models can be used to describe the heterogeneity in risk and also the differential impact of interventions not addressing this heterogeneity in risk [106, 107]. These models are defined by how the underlying processes (i.e. mechanisms) of transmission, disease progression (transitions between health-states), and/or an intervention’s causal effects are simulated. As such, these models capture the downstream impact of prevention among populations at highest risk of acquisition and transmission [108]. Common data inputs for these models include population size estimates, sexual behavior data, as well as biological parameters, and rates of access to interventions [62]. Data may be stratified so that they are reflective of heterogeneity in risks, including strata defined by age, sexual risk, and other population-level attributes. These models can then be used to estimate important outputs to guide HIV interventions and resource allocation, including stratified estimates of intervention coverage, HIV incidence and prevalence, and other related health outcomes [109, 110]. However, the model outputs are highly dependent on the parameters and model calibration to empirical estimates of model outputs, also known as “calibration targets”. Efforts can be made to reflect and address information and selection biases in these models to minimize the risks of the perpetuation of inequities. For example, the duration and relative infectivity of acute phase HIV can be sampled from wide prior distributions [111]. Alternatively, the modeled population can be stratified into subgroups comprising those who have “never tested” or “ever tested” for HIV, with the idea of capturing a testing gap across more marginalized populations that would be invisible in an “average testing rate” [109]. Similarly, the proportion of highly mobile populations who are captured in household-based HIV prevalence data can be explicitly modeled [112].

Model calibration is an integral step in applied modeling that precedes counterfactual analyses. All too often the details of this laborious process are buried in the supplementary materials of peer-reviewed publications. Yet model calibration can offer stand-alone results, such as: the posterior (joint) distributions of model parameters, underlying transmission dynamics (“who infected whom”), and plausible short-term epidemic trajectories. Thus, a calibrated mathematical model reflects another framework for the synthesis of heterogeneous data, within a common set of modeling assumptions. Moving forward, including these complete details such as model design, parameterization, and calibration decisions, as well as exploring insights from the calibrated model, will be essential for ensuring transparency and accessibility in decision-making prior to analyses of counterfactual scenarios.

## Conclusions

The risk and burden of HIV are not evenly distributed anywhere in the world, including in the most broadly generalized HIV epidemic settings. It has become increasingly clear that understanding the distribution of these risks is central to comprehensively addressing the needs of communities with sustained risks for HIV acquisition and transmission. Big Data Science can help identify and address these needs, but only if guided by an approach that leads with equity. Equity has long been an afterthought in the context of the HIV response, but leveraging an equity-lens is necessary for mitigating the potential harms of traditional big data approaches related to privacy, data biases, and opportunity costs, while simultaneously leveraging new methodologies that maximize the utility of current data and resources. As described above, these methodologies can integrate multiple data sources through data cleaning, linkage, and validation; generate new insights from emerging data sources; fill data gaps and adjust for biases through predictive modeling; synthesize all available data through transmission modeling; estimate the impacts of addressing unmet needs through explanatory modeling; and identify efficient interventions through economic analysis. Ultimately, these methodologies will help fill data blind spots and capture risk heterogeneities and intervention gaps which continue to shape the epidemic. Given declining resources for the HIV response globally, it is more important than ever to identify and comprehensively address the unmet needs of people at risk of and living with HIV.

## Data Availability

No datasets were generated or analysed during the current study.
